# Persistent secondary hyperparathyroidism caused by parathyromatosis and supernumerary parathyroid glands in a patient on haemodialysis

**DOI:** 10.1186/s12882-020-01917-3

**Published:** 2020-07-06

**Authors:** Jun Yang, Jun Zhang, Ning-hu Liu, Hao Liu, Meng-jie Dong

**Affiliations:** The Department of Nuclear Medicine, the First Affiliated Hospital, College of Medicine, Zhejiang University, 310003 Hangzhou, People’s Republic of China

**Keywords:** Persistent secondary hyperparathyroidism, Parathyromatosis, Supernumerary parathyroid glands, Haemodialysis

## Abstract

**Background:**

Secondary hyperparathyroidism (SHPT) is a common high-risk factor for mortality in end-stage renal disease, and parathyromatosis and supernumerary parathyroid glands are very rare causes of persistent SHPT. Preoperative diagnosis and removal of all hyperplastic parathyroid glands are challenging. We report a rare case of persistent SHPT due to parathyromatosis and supernumerary parathyroid glands and successful management by multiple imaging modalities.

**Case presentation:**

A 53-year-old Chinese woman on haemodialysis experienced discomfort due to itching and bone pain due to persistent SHPT after parathyroidectomy. The supernumerary parathyroid glands and parathyromatosis were detected by multiple imaging modalities, including ^99m^Tc-sestamibi (^99m^Tc-MIBI) scans, ultrasonography and four-dimensional computed tomography (4D-CT) and then excised; pathological confirmation was performed. During follow-up, her serum calcium and parathyroid hormone levels were stable in the appropriate ranges, and no complications arose.

**Conclusions:**

Because of persistent SHPH after parathyroidectomy in patients with haemodialysis, multiple imaging modalities, including ^99m^Tc-MIBI scans, 4D-CT and ultrasonography, are helpful for detecting supernumerary parathyroid glands and parathyromatoses. Accurate preoperative localization of this rare lesion is important for management, enabling the removal of all affected parathyroid tissues.

## Background

Secondary hyperparathyroidism (SHPT), a common and serious complication of end-stage renal disease (ESRD), is characterized by increased parathyroid hormone (PTH), calcium and phosphate levels, with substantial mortality [1, 2]. Surgical parathyroidectomy is crucial for patients who are resistant to medical treatment [2]. Furthermore, resection of all hyperplastic parathyroid glands is important, as remnant parathyroid glands can be continuously stimulated, leading to persistent or recurrent SHPT, which occurs in 0.4–25% of cases [3, 4]. Persistent or recurrent SHPT is attributed to various causes, including failure to identify or entirely remove hyperplastic parathyroid, ectopic, supernumerary parathyroid glands and, very rarely, parathyromatosis [5–8]. Parathyromatosis is very rare and is defined as multiple nests of hyperfunctioning parathyroid tissue [9]. Preoperative diagnosis and subsequent treatment are challenging. We report a case of persistent SHPT after parathyroidectomy due to parathyromatosis and supernumerary parathyroid glands in a patient with haemodialysis. SHPT was discovered by multiple imaging modalities and successfully managed.

## Case presentation

**Fig. 1:**
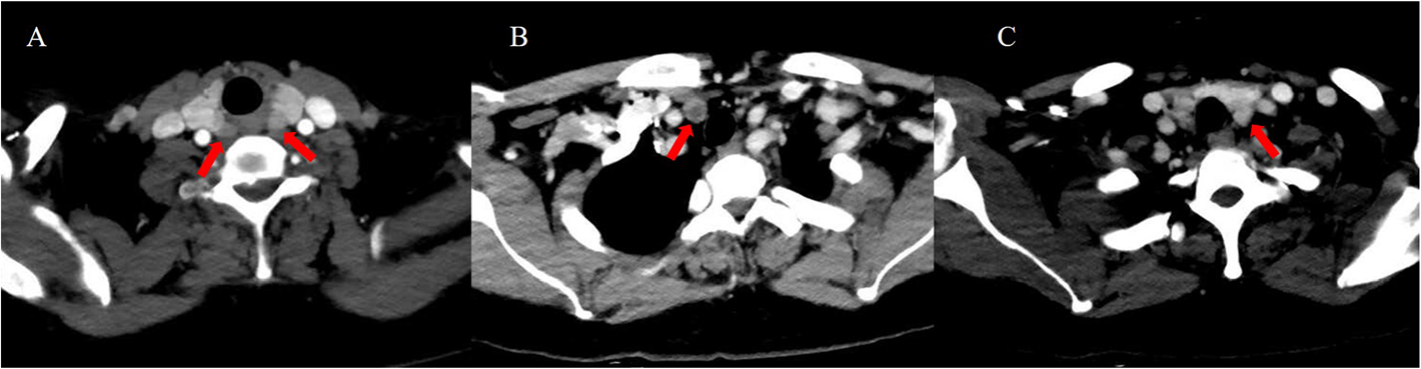
Axial arterial-phase computer tomography images (A-C) demonstrate four enhanced orthotopic parathyroid glands (the arrows) located posterior or inferior to the thyroid gland

**Fig. 2:**
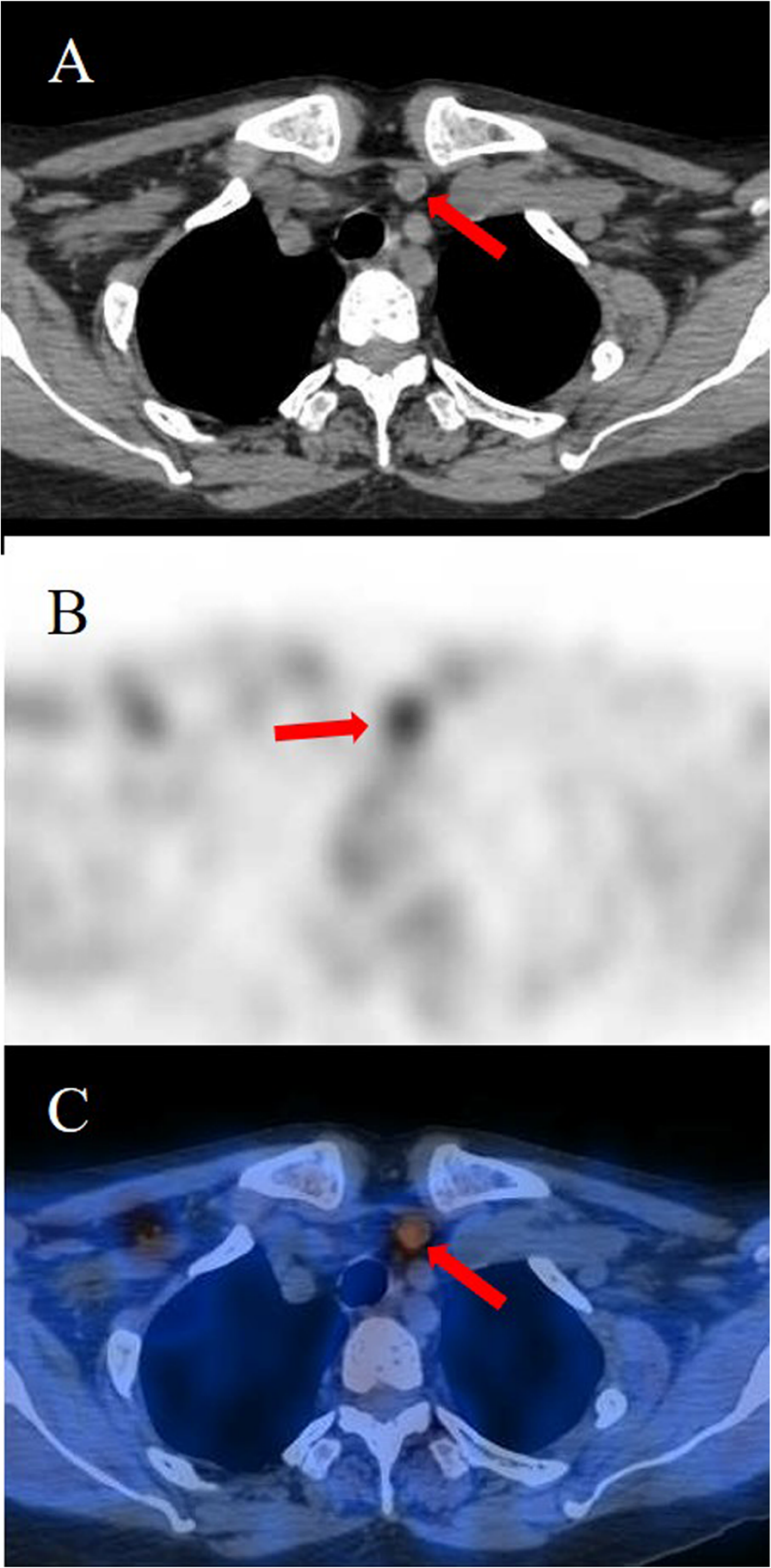
The ^99m^Tc-MIBI SPECT/CT image indicates a low-density lesion with high tracer uptake at the site of the inferior clavicle head (red arrows: A, axial image; B, SPECT; C, fusion image)

**Fig. 3:**
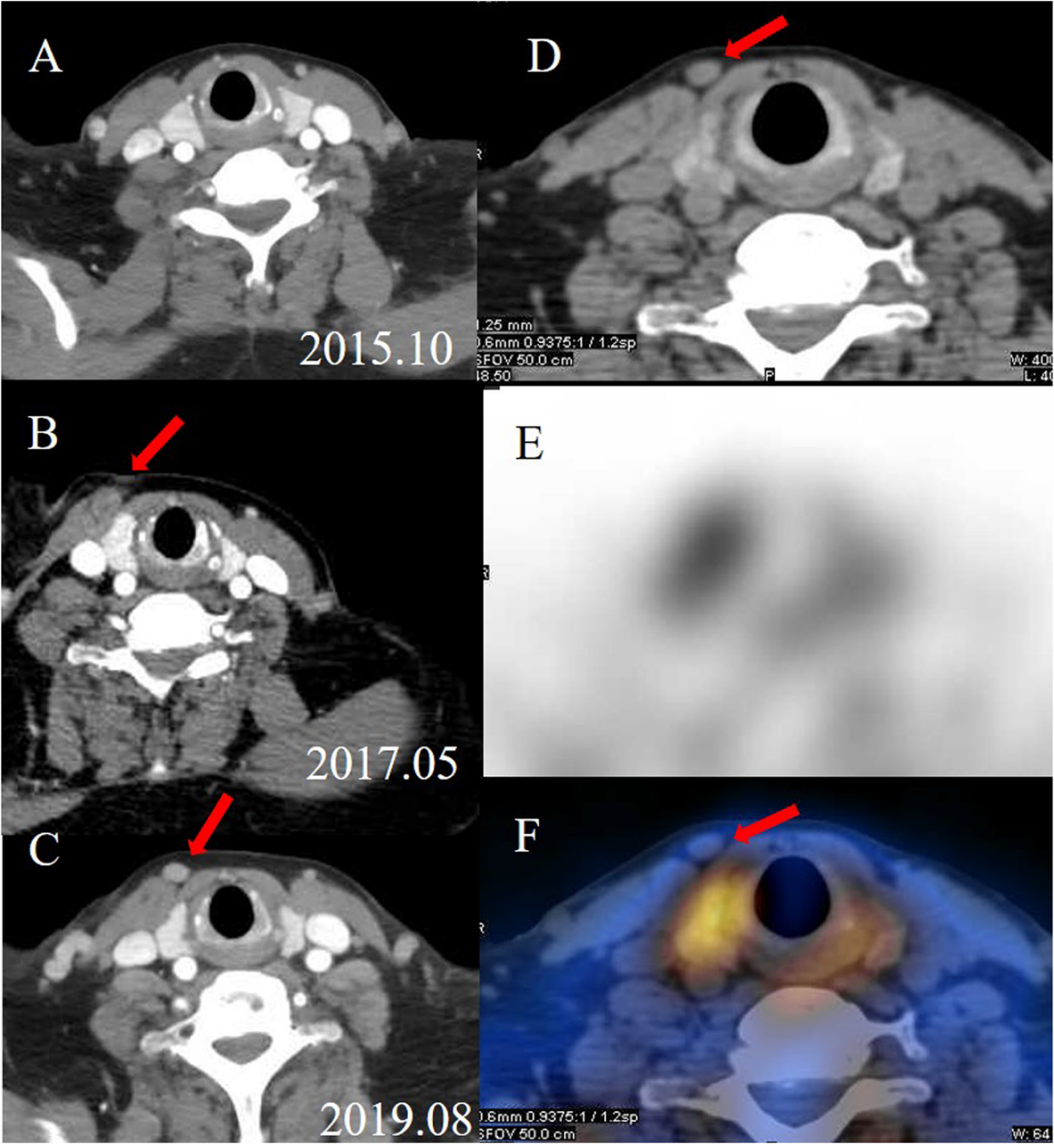
Serial arterial-phase axial 4D-CT of the parathyroid shows the growth of the right nodule (red arrows) located subcutaneously in the anterior sternocleidomastoid muscle (A, pre-operative; B, 18 months after surgery; C, 46 months after surgery). The ^99m^Tc-MIBI SPECT/CT image reveals the lesion without tracer uptake (red arrows: D, axial image; E, SPECT; F, fusion image)

**Fig. 4:**
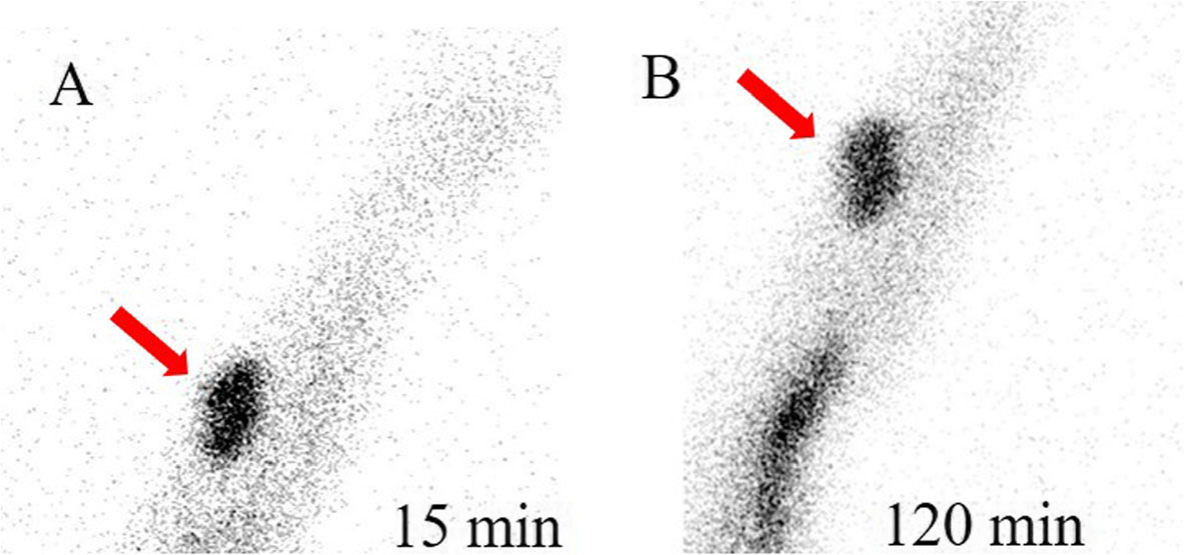
^99m^Tc-MIBI planar early (A) and delayed (B) static images demonstrate focal tracer accumulation (red arrows) at the site of autograft

**Fig. 5:**
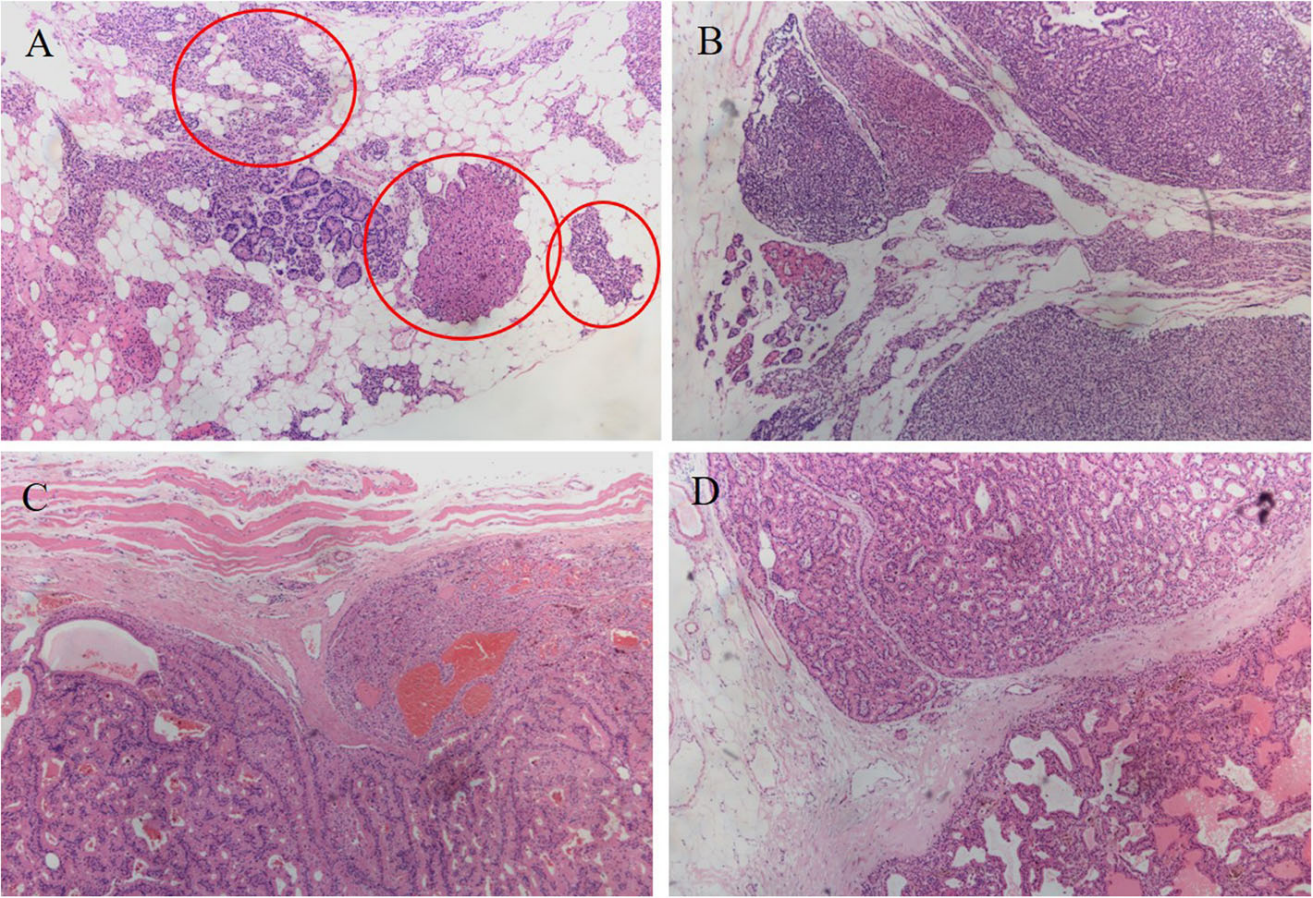
Histopathology (haematoxylin-eosin staining× 50) reveals hyperplastic parathyroid tissues composed of chief cells (A, red circles: microscopic nests of hypercellular parathyroid in the right inferior neck adipose tissue; B, supernumerary parathyroid gland; C nodule located in right forearm; D, subcutaneous nodule located anterior sternocleidomastoid muscle)

A 53-year-old Chinese woman had ESDR secondary to obstructive kidney disease and was treated with haemodialysis three times a week regularly since October 2008. She received a living donor kidney graft in May 2008 that was lost 5 months later because of rejection. SHPT was diagnosed based on elevated PTH and hypercalcaemia in July 2014. Although she had taken a vitamin D analogue (calcitriol and caltrate) and phosphate binder (lanthanum carbonate), her PTH and serum calcium levels gradually increased. Since October 2015, the patient had constantly experienced generalized itching. The results of the laboratory tests were as follows (normal ranges in parentheses): serum PTH, 942 pg/mL (10–65); serum calcium, 2.64 mmol/L (2.03–2.54); serum phosphate, 3.17 mmol/L (0.87–1.45); serum alkaline phosphatase, 77 U/L (40–150). Four-dimensional computed tomography (4D-CT, which originates from the number of phases of imaging, typically 3, and the change over time, the fourth dimension) of the parathyroid demonstrated four enhanced nodules consistent with orthotopic parathyroid glands (Fig. 1). No remarkable signs of ectopic or supernumerary parathyroid glands were observed. In November 2015, parathyroidectomy was performed to remove the four parathyroid glands, and the right inferior parathyroid gland was transplanted into the nondominant right forearm. On the first postoperative day, tests revealed PTH of 212 pg/mL, serum calcium of 2.43 mmol/L, and serum phosphate of 1.44 mmol/L, with alleviation of itching. Pathological findings showed parathyroid nodular hyperplasia without evidence of malignancy. Eighteen months after the surgery, she presented with recurred itching and bone pain. Serum calcium and PTH levels were 2.56 mmol/L and 1631 pg/mL, respectively. Planar ^99m^Tc-sestamibi (^99m^Tc-MIBI) scanning failed to localize the lesion. However, ultrasonography identified a remnant parathyroid gland in the left inferior thyroid, and she subsequently underwent percutaneous ethanol injection therapy (PEIT). On the third day of PEIT, her PTH and serum calcium levels decreased to 447 pg/mL and 2.15 mmol/L, respectively. In August 2019, the patient again experienced itching and bone pain and was admitted due to elevated serum calcium of 3.08 mmol/L and an elevated PTH level of 1593 pg/mL. Physical examination revealed a well-healed cervical surgical scar without a palpable mass. ^99m^Tc-MIBI single-photon emission computed tomography/computer tomography (SPECT/CT) indicated focal tracer uptake in the left inferior clavicle head (Fig. 2), consistent with the findings of 4D-CT and ultrasonography. A right nodule without tracer uptake was located subcutaneously in the anterior sternocleidomastoid muscle, which was also identified by serial 4D-CT (Fig. 3). A ^99m^Tc-MIBI planar scan also demonstrated focal tracer uptake in the autografted site in the early and delayed phases (Fig. 4), accompanied by a hypoechoic mass on ultrasonography. Three nodules were identified during bilateral neck exploration and then removed (the right subcutaneous nodule, right inferior and left inferior clavicle head were 1.0 × 0.8 cm, 0.6 × 0.5 cm and 1.5 × 1.0 cm, respectively). On the first day after the operation, the PTH level in a blood specimen obtained from the right arm was 11,226 pg/mL, whereas it was 1029 pg/mL from the left arm. In September 2019, surgery to remove the nodules in the autografted site was performed. The next day, her PTH level had decreased to 6.9 pg/mL. Pathological results showed all four nodules to be hyperplastic parathyroid, with several small nodular foci of atypical hyperplastic parathyroid tissue in the fat adjacent to the right inferior remnant parathyroid (Fig. 5). According to the clinical course and pathological findings, parathyromatosis and supernumerary parathyroid glands were confirmed. During 6 months of follow-up, her itching and bone pain improved, and she exhibited appropriate calcium (2.04–2.38 mmol/L) and PTH (84.4–145.0 pg/mL) levels under regular haemodialysis (Table 1).

**Table 1:** Changes in laboratory values in response to therapeutic interventions during the last 6 years

Time	Treatment	Calcium	Phosphorus	Parathyroid hormone
July 2014	Calcitriol + caltrate +lanthanum carbonate	2.43	1.58	171
October 2015	Before parathyroidectomy	2.64	3.17	942
November 2015	One day after parathyroidectomy + right forearm autograft	2.43	1.44	212
May 2017	Before percutaneous ethanal injection therapy	2.56	2.16	1631
	Three days after percutaneous ethanal injection therapy	2.15	1.58	447
August 2019	Before bilateral neck exploration	3.08	2.68	1593
September 2019	Before right forearm autograft resection	2.58	1.68	11,226 (right arm) 1029 (left arm)
	One day after right forearm autograft resection and	2.17	1.25	6.9
During 6 months of follow-up	regular haemodialysis	2.04–2.38	1.01–1.35	84.4–145.0

Calcium (mmol/L, normal 2.03–2.54); Phosphorus (mmol/L, normal 0.87–1.45); Parathyroid hormone (pg/mL, normal 10–65)

## Discussion and conclusions

Parathyromatosis and supernumerary parathyroid glands are very rare causes of persistent SHPT after parathyroidectomy in patients undergoing haemodialysis. The form of parathyromatosis is speculated to be either the spillage and seeding of the parathyroid tissue during parathyroid surgery (type 2) or the development of embryonic rests of parathyroid tissue under physiological stimuli (type 1) [9–12]. In our case, one of the causes of the persistent hyperparathyroidism was considered to be type 2 parathyromatosis because both implanted autografts in the forearm and un-implanted subcutaneous seeding were observed. The existing factor of metabolic derangements in ESRD continues to stimulate the growth of implants [13]. Different stages of parathyroid hyperplasia may exist in the same patient because of the heterogeneity of the parathyroid glands with regard to different expression of calcium-sensing receptors, even under the same stimuli. We considered that rupture of the capsule may have occurred during the first parathyroidectomy, as a growth in the right subcutaneous anterior sternocleidomastoid muscle was revealed during serial 4D-CT. According to the literature, most cases are female subjects with ESRD in their fifth to sixth decade of life, which fits the profile of our patient [14]. The other cause of persistent hyperparathyroidism is the supernumerary parathyroid glands, which were overlooked in situ during the first operation; they were located in the left inferior clavicle head. Supernumerary parathyroid glands, which are found in approximately 13% of autopsy and 30–39% of re-operative cases [5, 15–17], may result from separation of the parathyroid anlage during embryologic migration, and most glands are located in the thymus or superior mediastinum [18]. Knowledge of the anatomy and embryology of parathyroid glands and meticulous handling of parathyroid glands to avoid rupture and spillage during surgery are essential for successful parathyroidectomy. Although complete surgical extirpation of all offending parathyroid nodules is the best option for patients with haemodialysis, it often does not occur [7]. Indeed, the greatest difficulty lies in precise preoperative localization and removal of all disseminated tiny and unexpected nodules of parathyroid tissue [14]. Preoperative imaging can help to identify supernumerary glands and parathyromatosis, but only 40% of patients are preoperatively diagnosed [7]. Moreover, there is no universally accepted consensus for preoperative localization, as different imaging modalities (ultrasonography, ^99m^Tc-MIBI SPECT/CT, 4D-CT) have various advantages and disadvantages. Depending on the clinical scenario, physicians should choose a suitable image modality. At our institution, US and ^99m^Tc-MIBI SPECT/CT are the first-line modalities for re-operation location of parathyroid lesions. When the first-line imaging modalities are negative or discordant, 4D-CT or MRI is used. Fortunately, all of the affected parathyroid nodules were successfully identified and managed in our patient, as also occurred in the rare accounts in the literature [18–20]. Ultrasonography can sometimes identify scattered hypoechoic, hypervascular cervical nodules that appear nonspecific and do not conform to typical anatomic locations of the parathyroid glands [8]. Nonetheless, this modality is operator dependent and unable to assess mediastinal sites, with difficulty in imaging of far-posterior lesions [21]. Overall, ^99m^Tc-MIBI SPECT/CT can provide both functional and anatomic information and improve the detection of ectopic/supernumerary glands or far-posterior lesions, which ultrasonography is prone to miss. This technique can also reveal uptake foci and has superior (95%) positive predictive value [22]. Similar to other studies, some of the lesions in our patients were localized by ^99m^Tc-MIBI SPECT/CT [5, 19, 21, 23]. 4D-CT is a relatively new imaging modality that is characterized by unique perfusion. In a study of 58 patients who had recurrent hyperparathyroidism, the sensitivity of localization of hyperfunctioning parathyroid tissue was 77.4% for 4D-CT compared to 46.0% for ^99m^Tc-MIBI imaging [24]. In our study, serial 4D-CT located the supernumerary parathyroid glands and revealed the growth of parathyromatosis in the subcutaneous anterior sternocleidomastoid muscle in the patient, which was consistent with the literature [18, 21, 24, 25].

Regardless, the treatment and control of supernumerary parathyroid glands and parathyromatosis remain a challenge, as complete excision of all foci is very difficult. When multiple surgeries fail, medical treatment, including suppression of PTH production with cinacalcet and stabilization of bone mineral density with bisphosphonate, should be considered [8, 14, 26]. In conclusion, parathyromatosis and supernumerary parathyroid glands are very rare causes of persistent hyperparathyroidism in patients undergoing haemodialysis. Multiple imaging modalities are valuable for precise preoperative localization of affected parathyroid tissues and may help with subsequent treatment.

## Data Availability

Not applicable.

## Abbreviations

SHPT: Secondary hyperparathyroidism
^99m^Tc-MIBI: ^99m^Tc-sestamibi
4D-CT: Four-dimensional computed tomography
ESRD: End-stage renal disease
SPECT/CT: Single-photon emission computed tomography/computer tomography
